# Supplementary material for: Are the Blueberries We Buy Good Quality? Comparative Study of Berries Purchased from Different Outlets
Source: Foods. 2023 Jul 6;12(13):2621. doi: 10.3390/foods12132621 (PMC10340143; doi:10.3390/foods12132621)

Supplementary material S1. Examples of chromatograms and MS spectrum results for standards and samples from different outlets. All measurements were performed in triplicate. Samples 1-3: Sale point 4 in the manuscript; samples 4-6: Sale point 3 in the manuscript; samples 7-9: Sale point 5 in the manuscript; samples 10-12: Sale point 1 in the manuscript; samples 13-15: Sale point 2 in the manuscript.

STANDARD MULTIPLE (CHLOROGENIC ACID, CUMARIC ACID, CAFFEIC ACID AND QUERCETIN)

CHLOROGENIC ACID STANDARD (355.10)

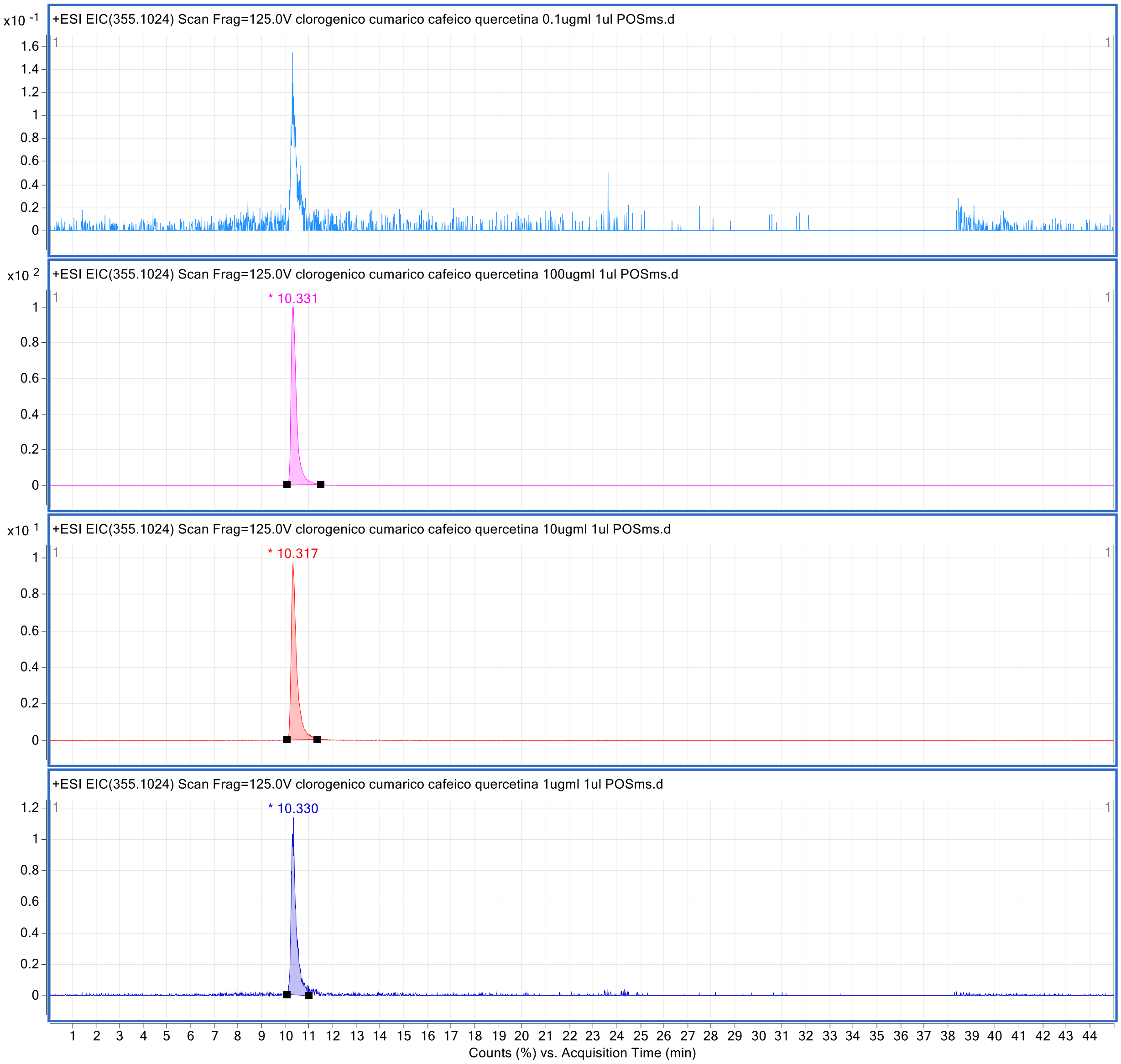

Best	ID Source	Name	Formula	Species	m/z	Score	Score (RT)	RT
◉	MFG		C16 H18 O9	(M+H)+	355.1029	99		
○	MFG		C15 H12 N7 O4	(M+H)+	355.1029	96.22		
○	MFG		C17 H14 N4 O5	(M+H)+	355.1029	95.75		
○	MFG		C14 H16 N3 O8	(M+H)+	355.1029	89.35		
○	MFG		C9 H18 N6 O7 S	(M+H)+	355.1029	87.05		
○	MFG		C10 H14 N10 O3 S	(M+H)+	355.1029	87.01		
○	MFG		C11 H20 N3 O8 S	(M+H)+	355.1029	85.99		
○	MFG		C13 H10 N10 O3	(M+H)+	355.1029	85.22		
○	MFG		C23 H16 N O S	(M+H)+	355.1029	84.44		
○	MFG		C19 H16 N O6	(M+H)+	355.1029	84.34		
○	MFG		C17 H22 O4 S2	(M+H)+	355.1029	83.39		
○	MFG		C18 H10 N8 O	(M+H)+	355.1029	81.84		
○	MFG		C15 H20 N3 O3 S2	(M+H)+	355.1029	78.47		
○	MFG		C18 H18 N4 S2	(M+H)+	355.1029	77.35		
○	MFG		C12 H16 N7 O4 S	(M+H)+	355.1029	77.23		
○	MFG		C21 H14 N4 S	(M+H)+	355.1029	76.75		
○	MFG		C13 H22 O9 S	(M+H)+	355.1029	75.05		
○	MFG		C12 H14 N6 O7	(M+H)+	355.1029	73.49		

Chromatogram Results | Spectrum Identification Results: + Scan (rt: 10.400 min)

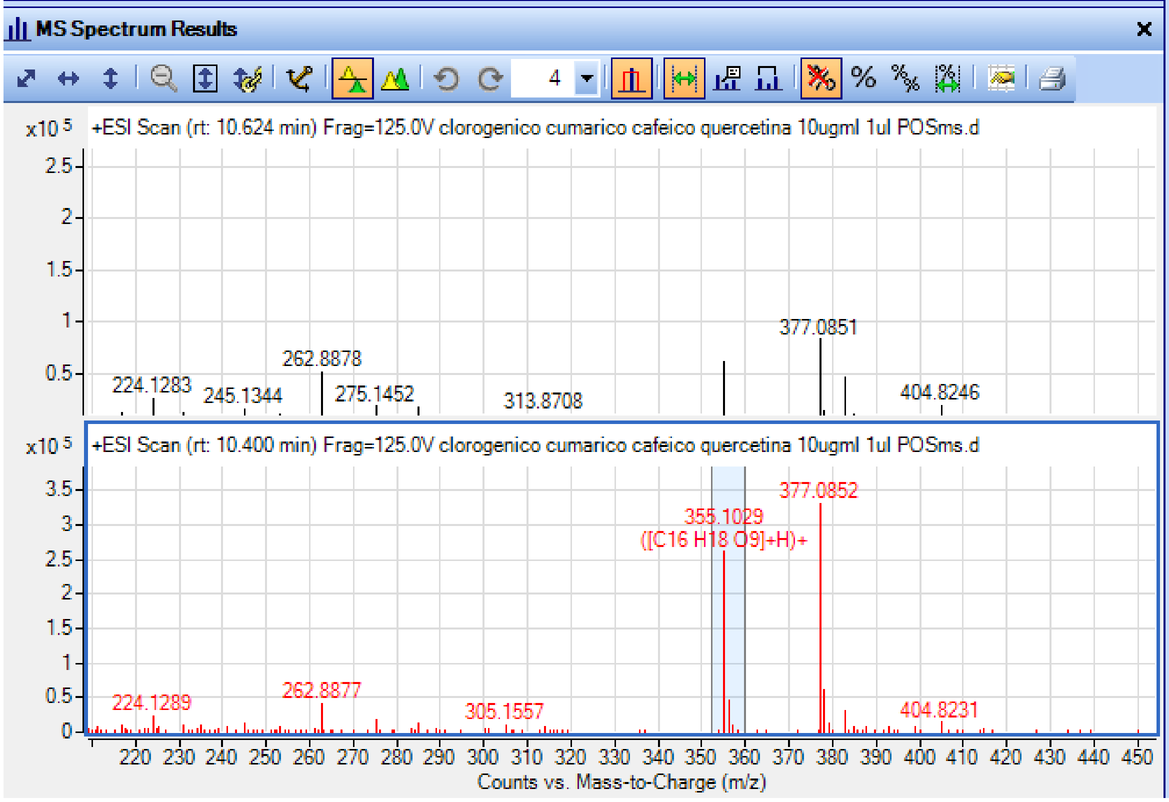

QUERCETIN-3-GLUCOSIDE (465.1028) AND QUERCETIN (302.0421) STANDARDS

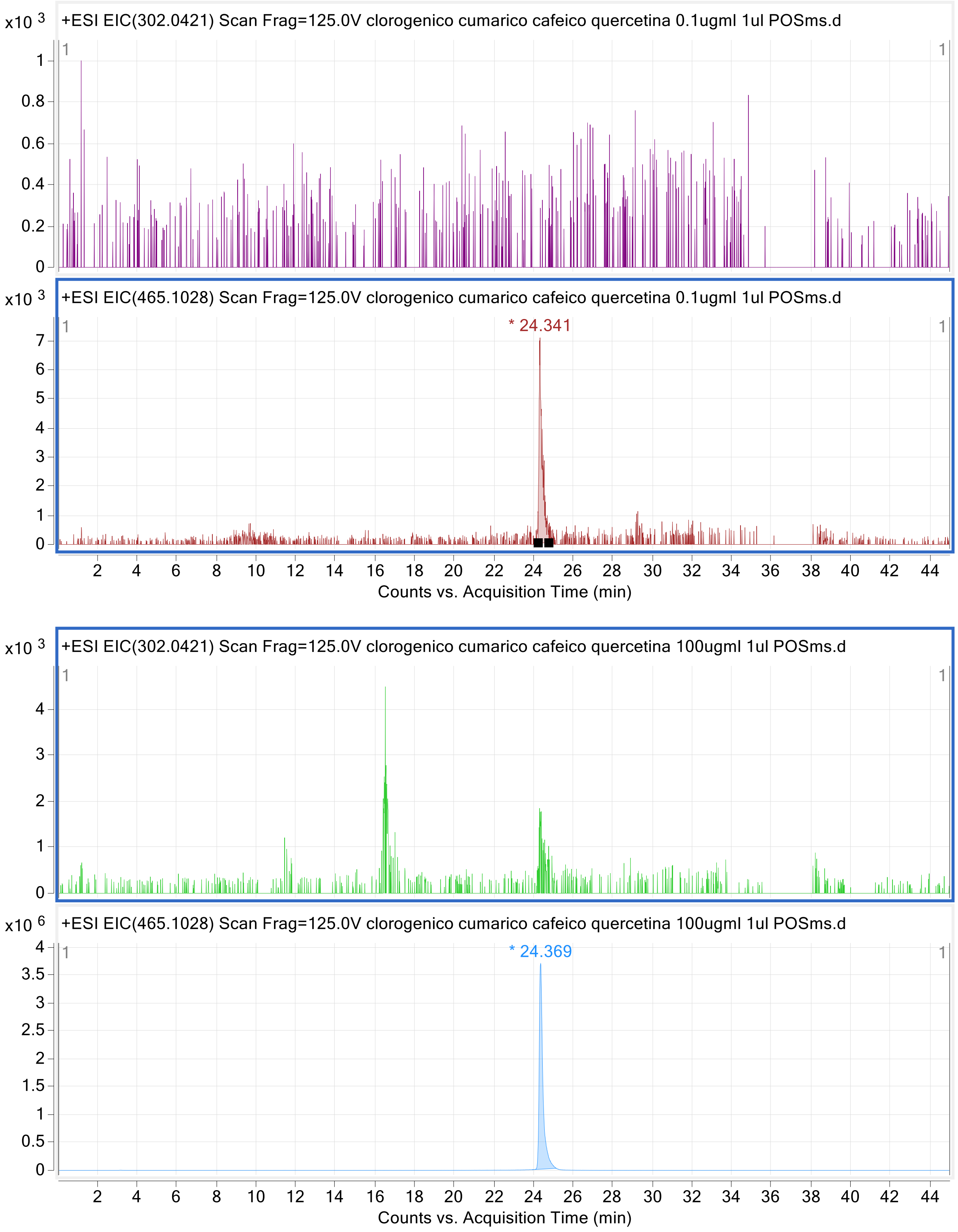

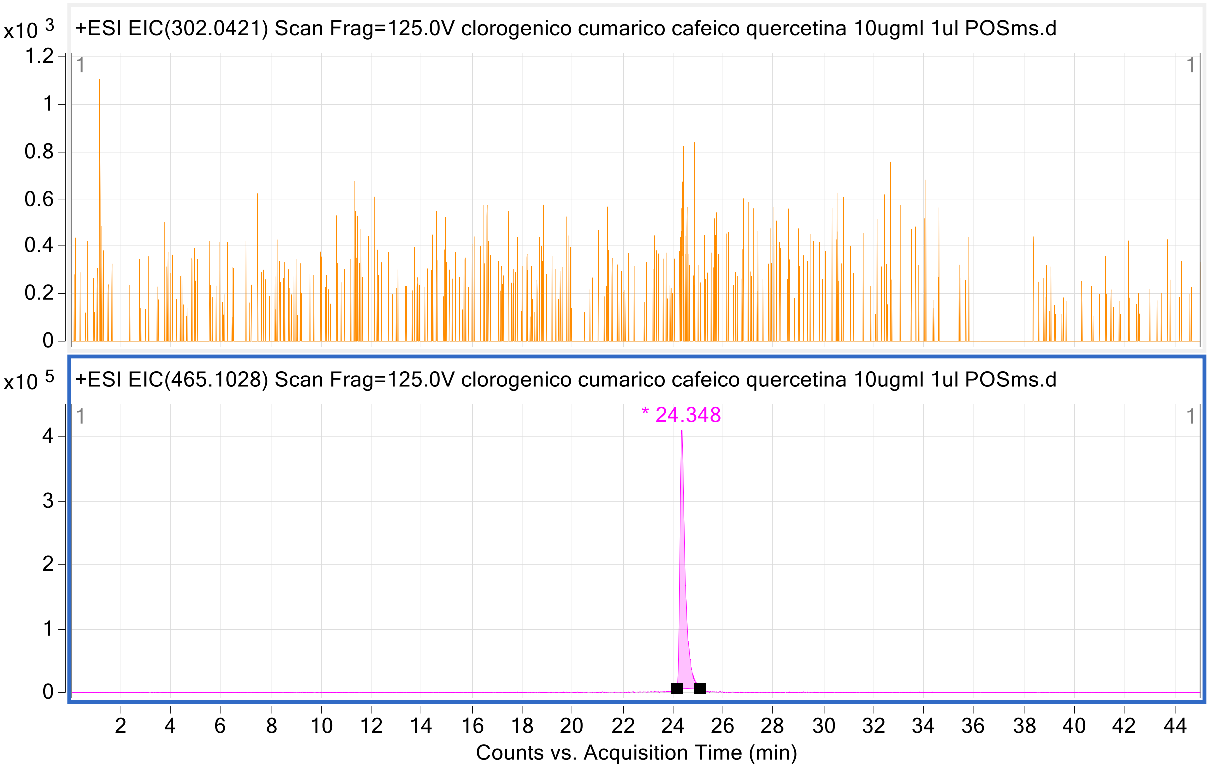
x10 3
+ESI EIC(302.0421) Scan Frag=125.0V clorogenico cumarico cafeico quercetina 10ugml 1ul POSms.d
x10 5
+ESI EIC(465.1028) Scan Frag=125.0V clorogenico cumarico cafeico quercetina 10ugml 1ul POSms.d
* 24.348
Counts vs. Acquisition Time (min)

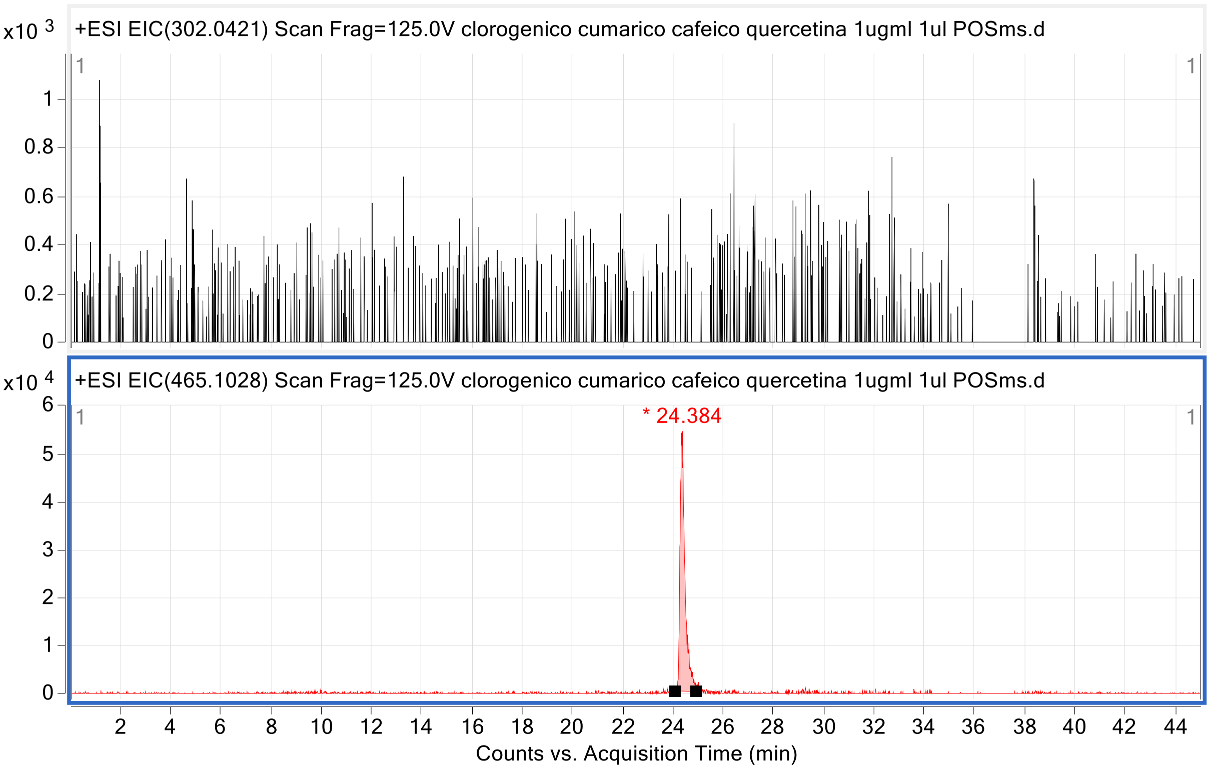
x10 3
+ESI EIC(302.0421) Scan Frag=125.0V clorogenico cumarico cafeico quercetina 1ugml 1ul POSms.d
x10 4
+ESI EIC(465.1028) Scan Frag=125.0V clorogenico cumarico cafeico quercetina 1ugml 1ul POSms.d
* 24.384
Counts vs. Acquisition Time (min)

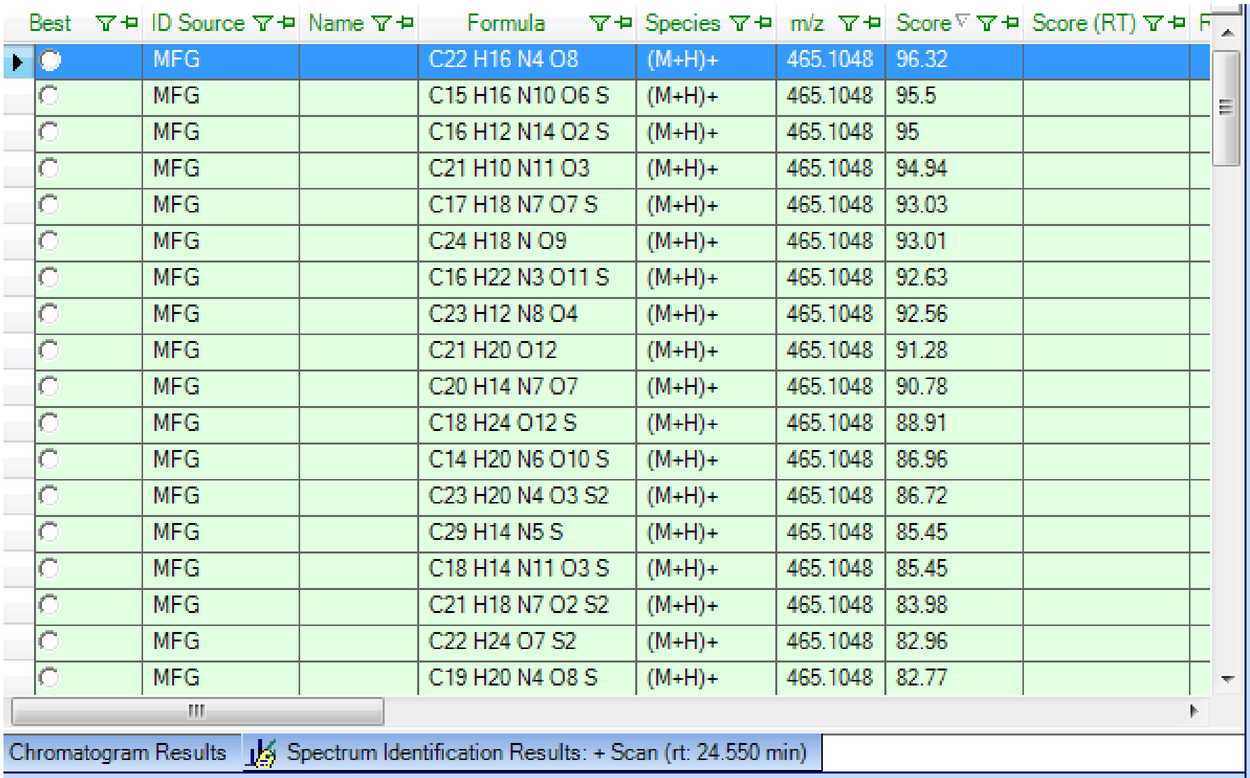

Best	ID Source	Name	Formula	Species	m/z	Score	Score (RT)	F
	MFG		C22 H16 N4 O8	(M+H)+	465.1048	96.32		
	MFG		C15 H16 N10 O6 S	(M+H)+	465.1048	95.5		
	MFG		C16 H12 N14 O2 S	(M+H)+	465.1048	95		
	MFG		C21 H10 N11 O3	(M+H)+	465.1048	94.94		
	MFG		C17 H18 N7 O7 S	(M+H)+	465.1048	93.03		
	MFG		C24 H18 N O9	(M+H)+	465.1048	93.01		
	MFG		C16 H22 N3 O11 S	(M+H)+	465.1048	92.63		
	MFG		C23 H12 N8 O4	(M+H)+	465.1048	92.56		
	MFG		C21 H20 O12	(M+H)+	465.1048	91.28		
	MFG		C20 H14 N7 O7	(M+H)+	465.1048	90.78		
	MFG		C18 H24 O12 S	(M+H)+	465.1048	88.91		
	MFG		C14 H20 N6 O10 S	(M+H)+	465.1048	86.96		
	MFG		C23 H20 N4 O3 S2	(M+H)+	465.1048	86.72		
	MFG		C29 H14 N5 S	(M+H)+	465.1048	85.45		
	MFG		C18 H14 N11 O3 S	(M+H)+	465.1048	85.45		
	MFG		C21 H18 N7 O2 S2	(M+H)+	465.1048	83.98		
	MFG		C22 H24 O7 S2	(M+H)+	465.1048	82.96		
	MFG		C19 H20 N4 O8 S	(M+H)+	465.1048	82.77		

Chromatogram Results | Spectrum Identification Results: + Scan (rt: 24.550 min)

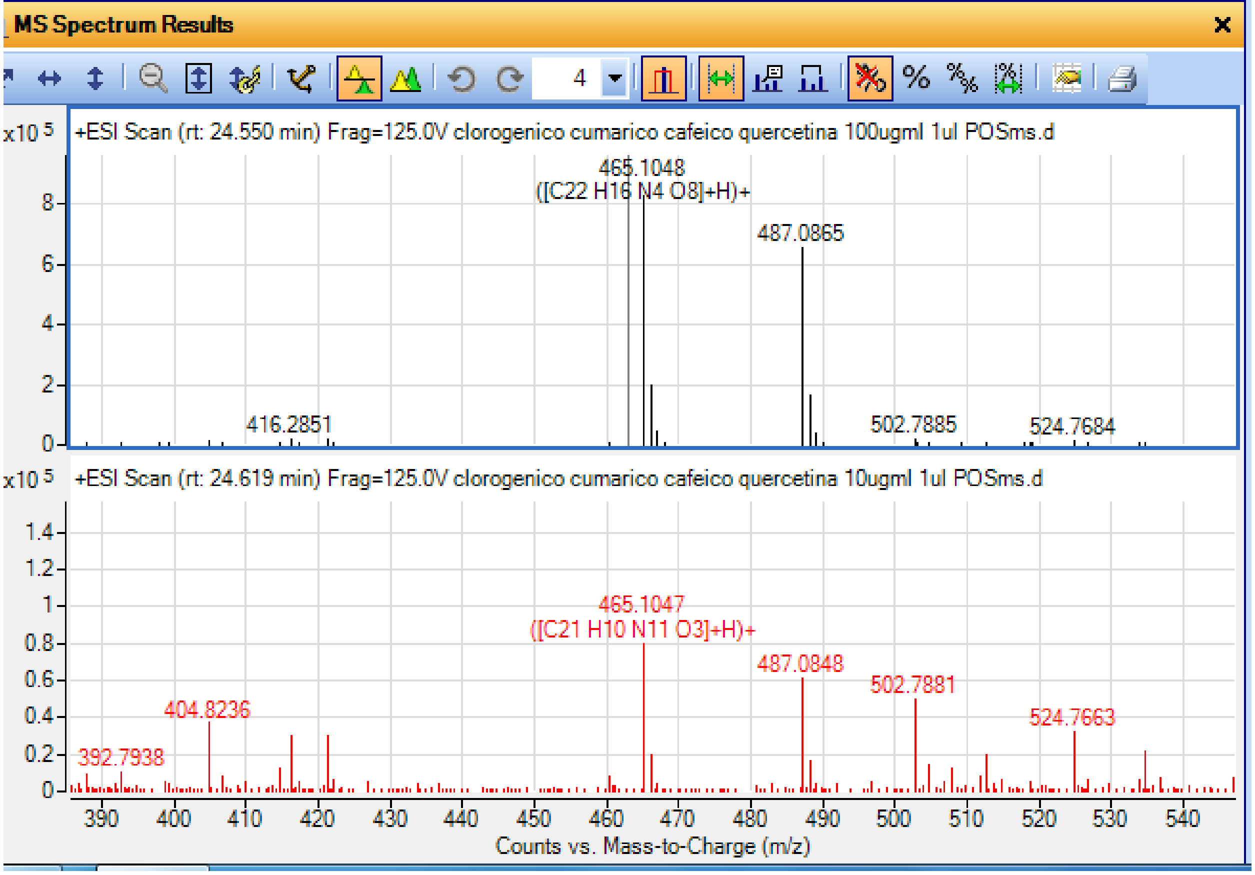

CHLOROGENIC ACID IN SAMPLES

SALE POINT 4 (TRIPLICATE)

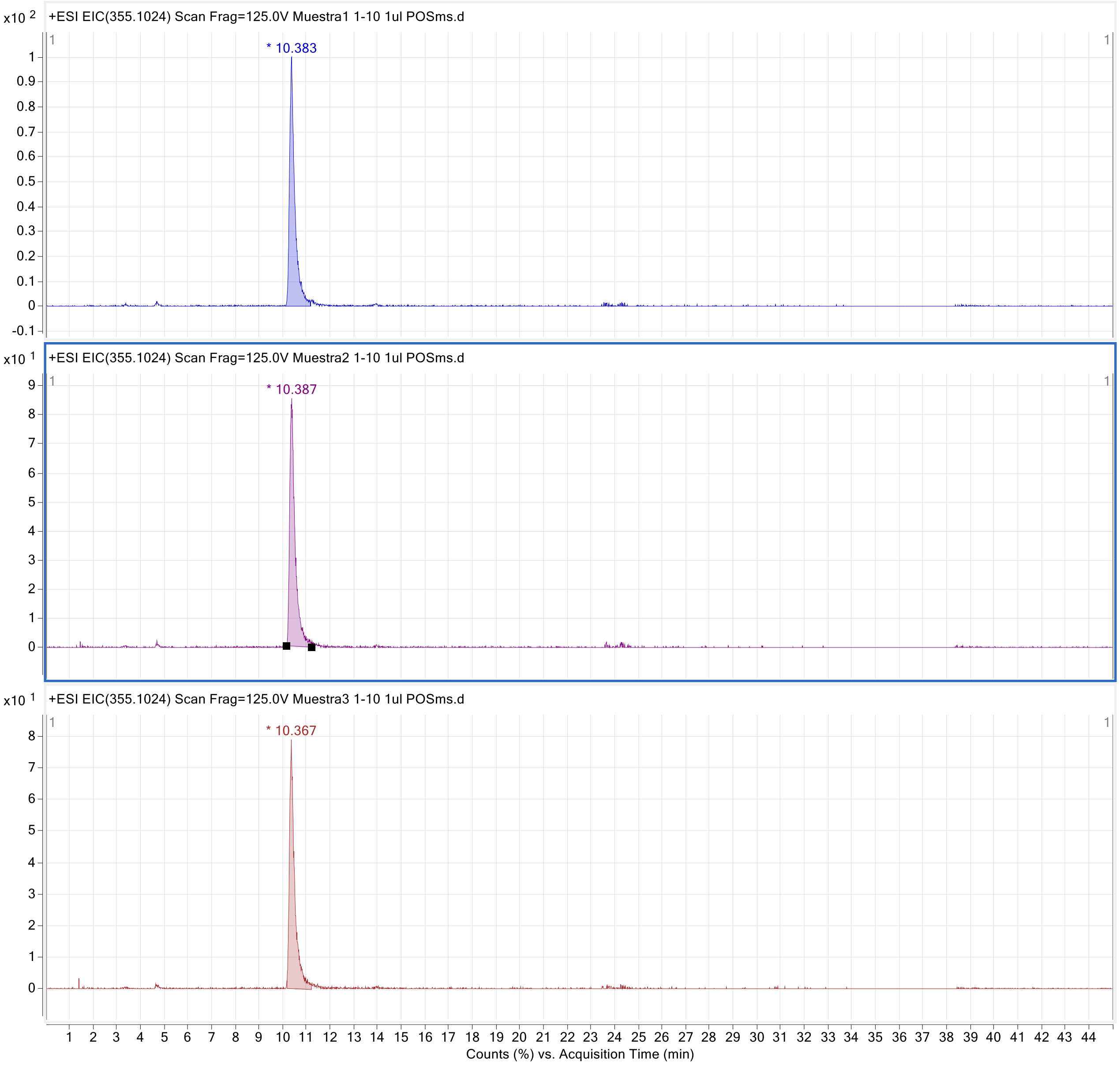

DELPHINIDIN 3-PYRANOSIDE (465.1028) IN SAMPLES

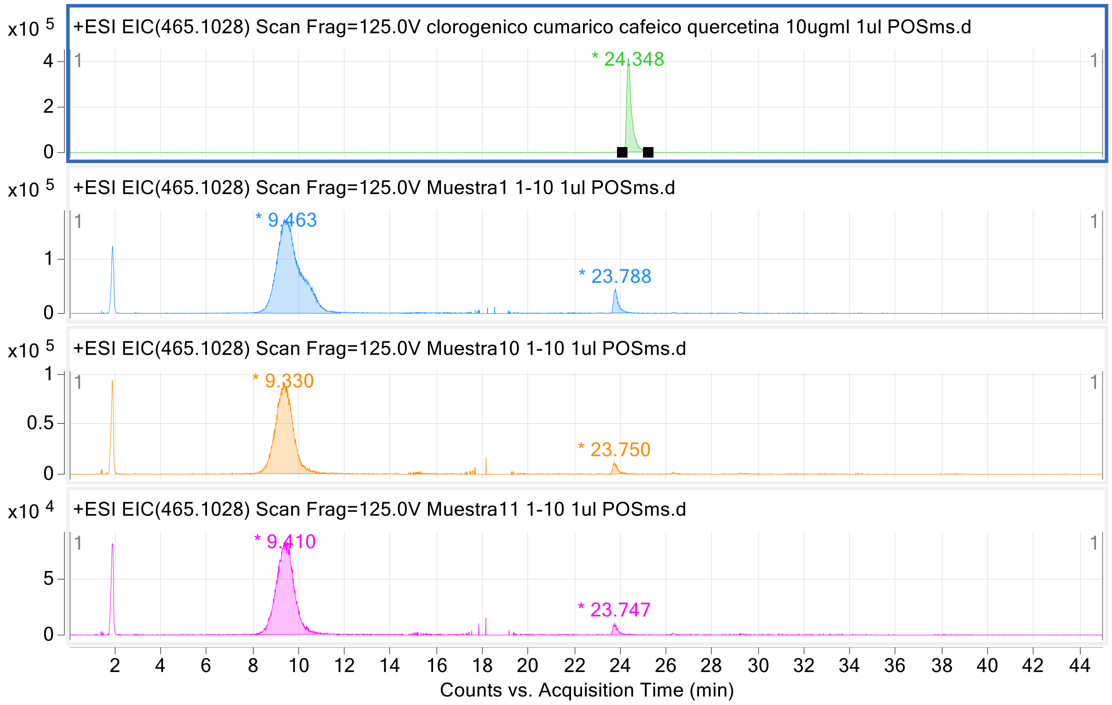

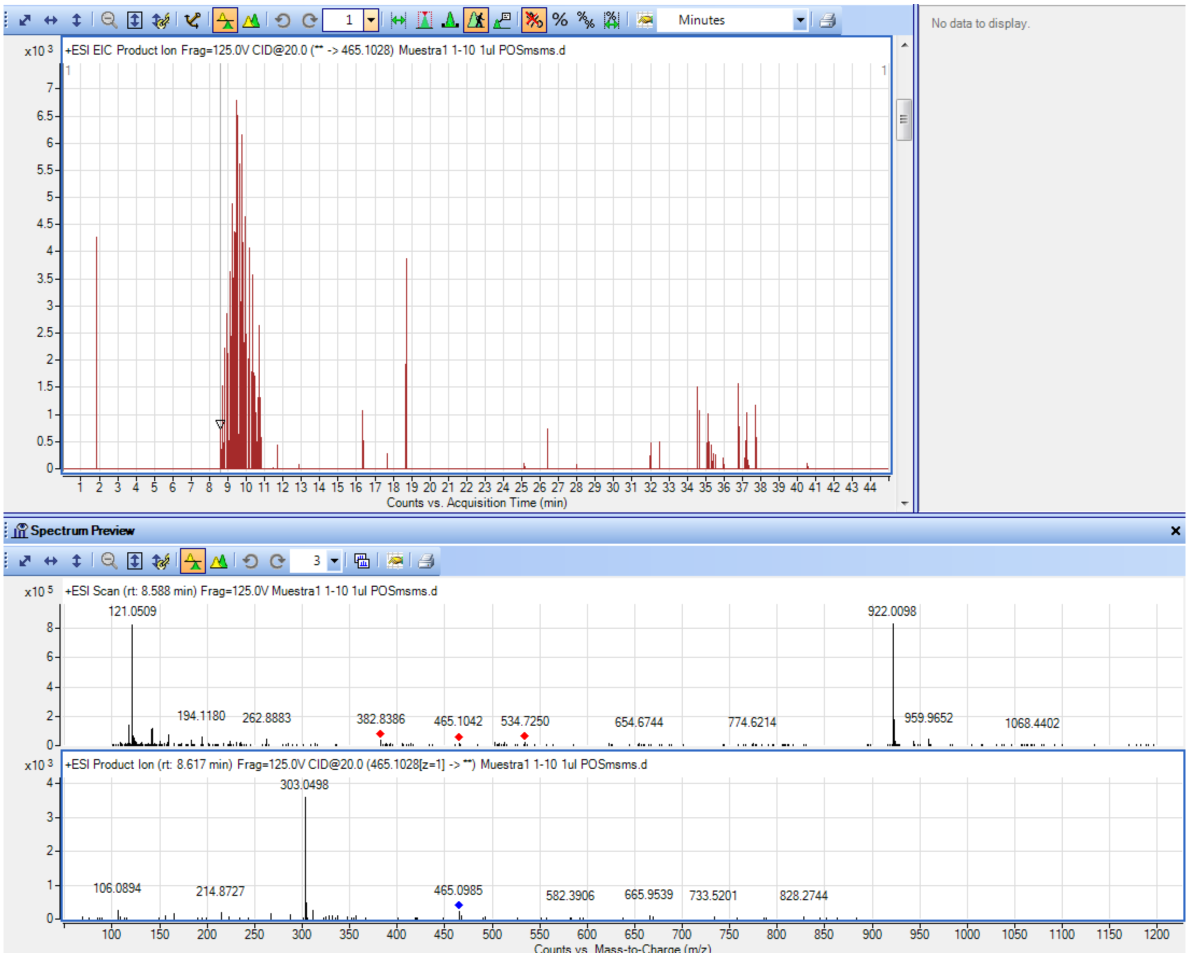

STANDARD SOLUTIONS

1. Malvidin-3-glucoside (493.1341)

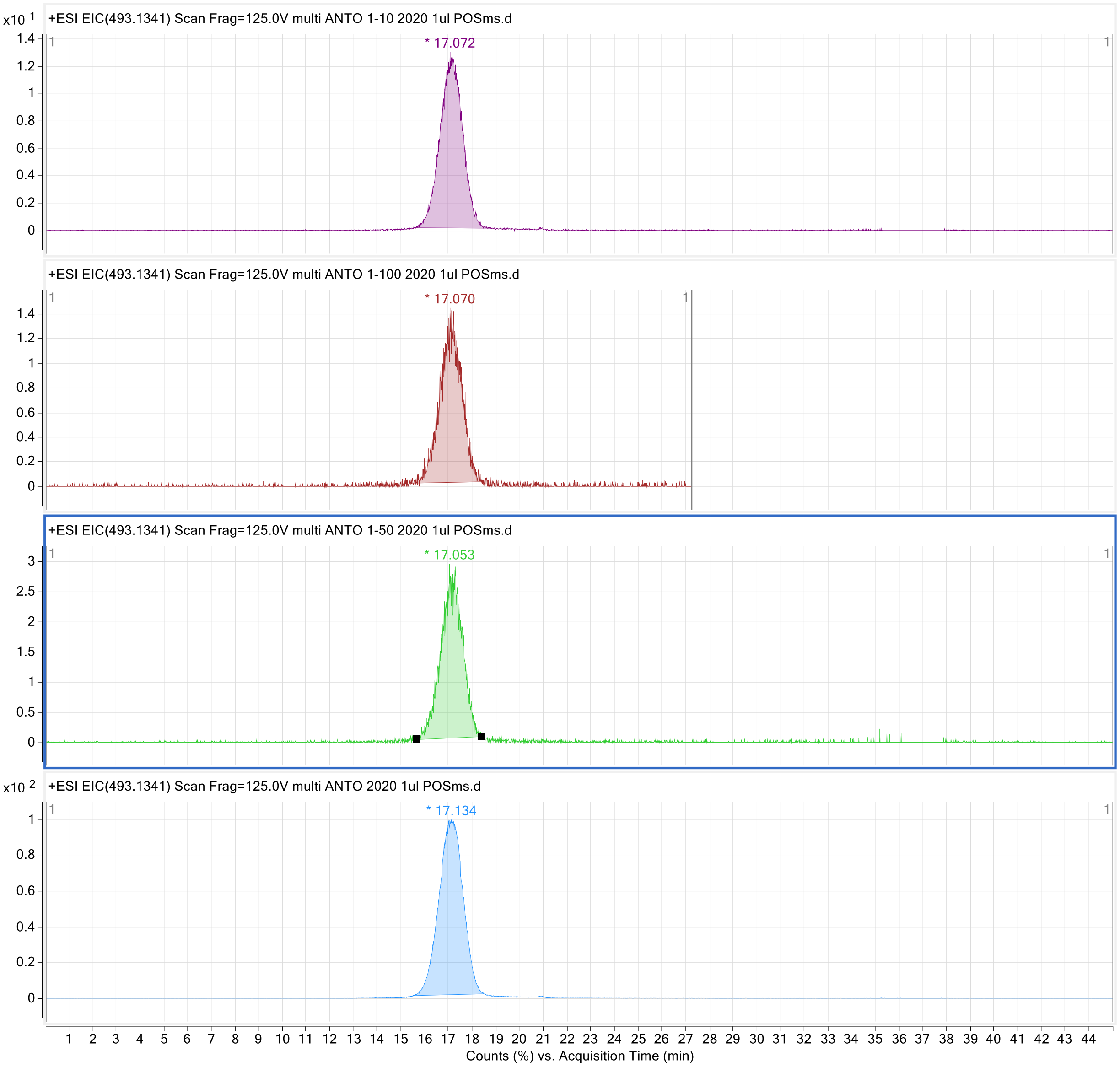

Best	ID Source	Name	Formula	Species	m/z	Score	Score (RT)	RT Diff	Diff (ppm)	Score (Lib
	MFG		C23 H24 O12	(M+H)+	493.1339	98.67			-0.06	
	MFG		C22 H18 N7 O7	(M+H)+	493.1339	98.09			-0.37	
	MFG		C24 H20 N4 O8	(M+H)+	493.1339	95.79			2.5	
	MFG		C21 H12 N14 O2	(M+H)+	493.1339	95.72			-0.69	
	MFG		C23 H14 N11 O3	(M+H)+	493.1339	94.36			2.2	
	MFG		C21 H22 N3 O11	(M+H)+	493.1339	93.17			-2.94	
	MFG		C20 H16 N10 O6	(M+H)+	493.1339	91.65			-3.27	
	MFG		C30 H22 N O4 S	(M+H)+	493.1339	89			-0.12	
	MFG		C26 H22 N O9	(M+H)+	493.1339	86.36			5.37	
	MFG		C28 H20 N4 O3 S	(M+H)+	493.1339	85.65			-2.98	
	MFG		C25 H16 N8 O4	(M+H)+	493.1339	85.47			5.08	
	MFG		C15 H18 N13 O5 S	(M+H)+	493.1339	84.52			0.1	
	MFG		C36 H16 N2 O	(M+H)+	493.1339	84.5			-1.09	
	MFG		C16 H24 N6 O10 S	(M+H)+	493.1339	83.28			0.48	
	MFG		C31 H18 N5 S	(M+H)+	493.1339	83.17			2.48	
	MFG		C17 H20 N10 O6 S	(M+H)+	493.1339	82.59			3.03	
	MFG		C23 H22 N7 O2 S2	(M+H)+	493.1339	81.9			0.74	
	MFG		C19 H20 N6 O10	(M+H)+	493.1339	80.9			-5.83	

Chromatogram Results | Spectrum Identification Results: + Scan (rt: 17.154 min)

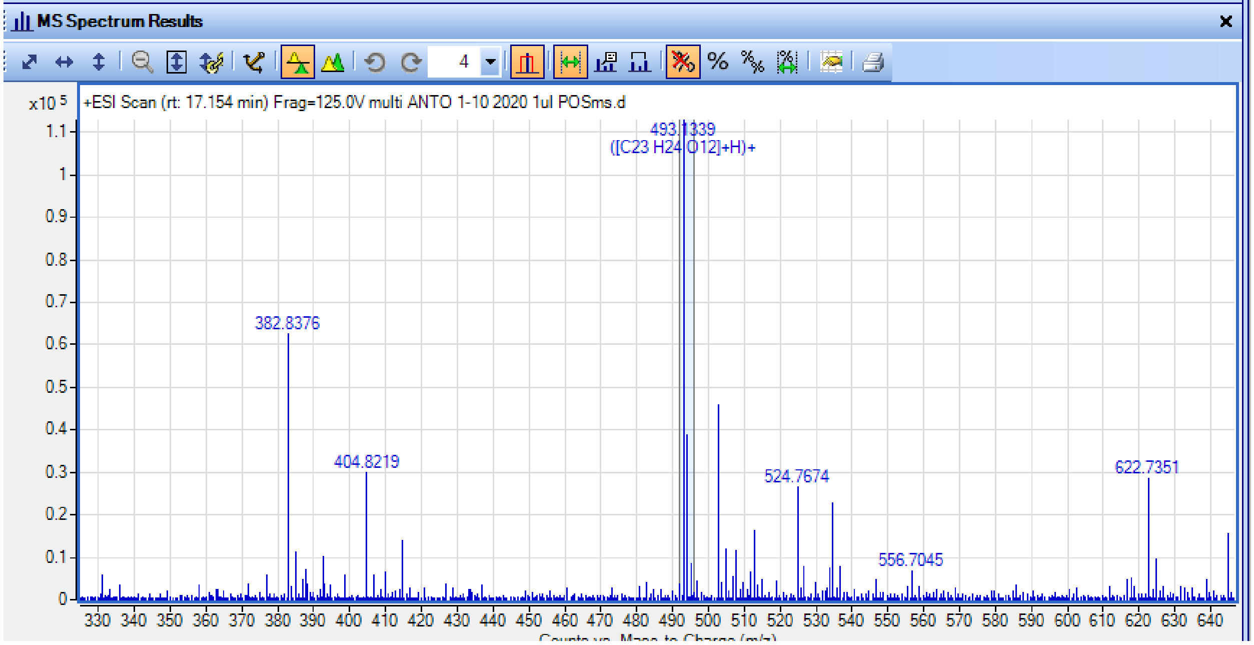

2. Cyanidin-3-glucoside (449.1078)

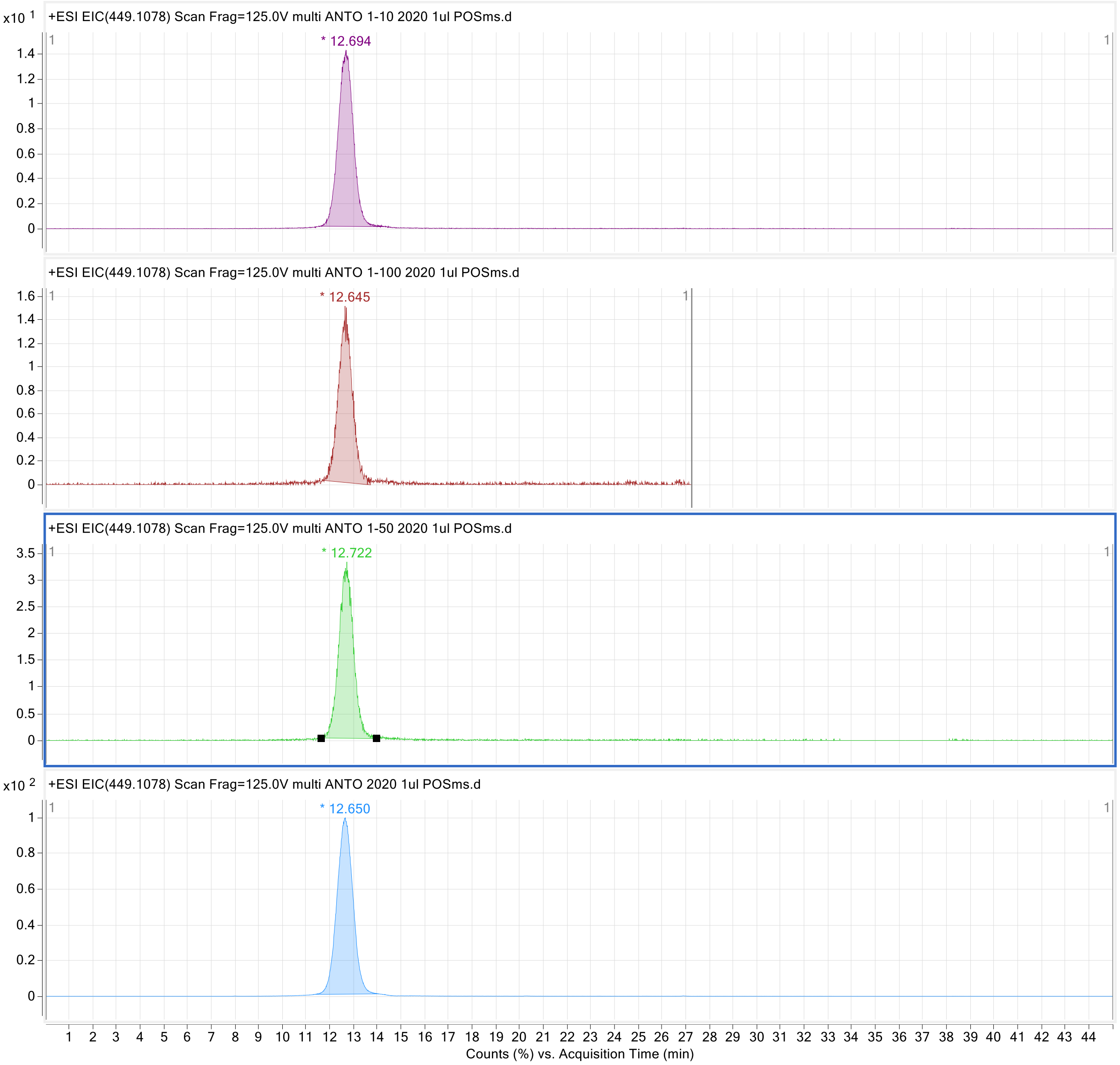

Automatically Show Columns

Best	ID Source	Name	Formula	Species	m/z	Score	Score (RT)	RT Diff	Diff (ppm)	Score (Lib
	MFG		C20 H14 N7 O6	(M+H)+	449.1083	97.88			-0.74	
	MFG		C21 H20 O11	(M+H)+	449.1083	97.68			-0.42	
	MFG		C22 H16 N4 O7	(M+H)+	449.1083	94.07			2.41	
	MFG		C21 H10 N11 O2	(M+H)+	449.1083	93.45			2.1	
	MFG		C19 H18 N3 O10	(M+H)+	449.1083	92.94			-3.57	
	MFG		C18 H12 N10 O5	(M+H)+	449.1083	92.32			-3.91	
	MFG		C14 H20 N6 O9 S	(M+H)+	449.1083	91.73			0.26	
	MFG		C15 H16 N10 O5 S	(M+H)+	449.1083	91.04			3.07	
	MFG		C16 H22 N3 O10 S	(M+H)+	449.1083	87.23			3.45	
	MFG		C28 H18 N O3 S	(M+H)+	449.1083	86.58			-0.44	
	MFG		C12 H18 N9 O8 S	(M+H)+	449.1083	86.35			-2.95	
	MFG		C21 H18 N7 O S2	(M+H)+	449.1083	85.74			0.58	
	MFG		C22 H24 O6 S2	(M+H)+	449.1083	83.99			0.88	
	MFG		C19 H16 N10 S2	(M+H)+	449.1083	83.9			-2.57	
	MFG		C13 H24 N2 O13 S	(M+H)+	449.1083	83.66			-2.52	
	MFG		C26 H16 N4 O2 S	(M+H)+	449.1083	83.21			-3.57	
	MFG		C20 H22 N3 O5 S2	(M+H)+	449.1083	83.05			-2.26	
	MFG		C16 H12 N14 O S	(M+H)+	449.1083	82.64			5.91	

Chromatogram Results | Spectrum Identification Results: + Scan (rt: 12.564 min)

MS Spectrum Results

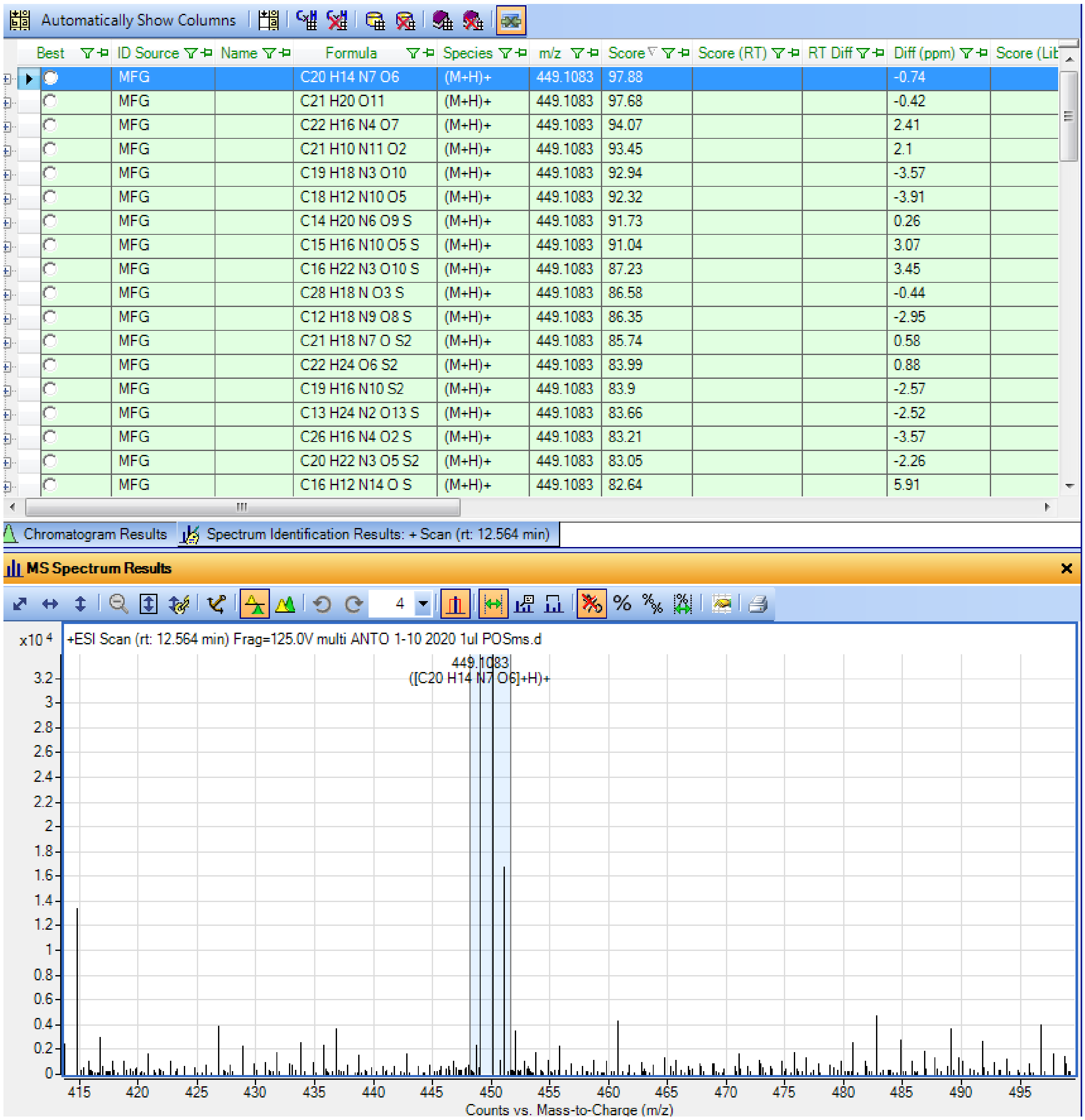

MALVIDIN 3-GLUCOSIDE (493.1341) IN SAMPLES

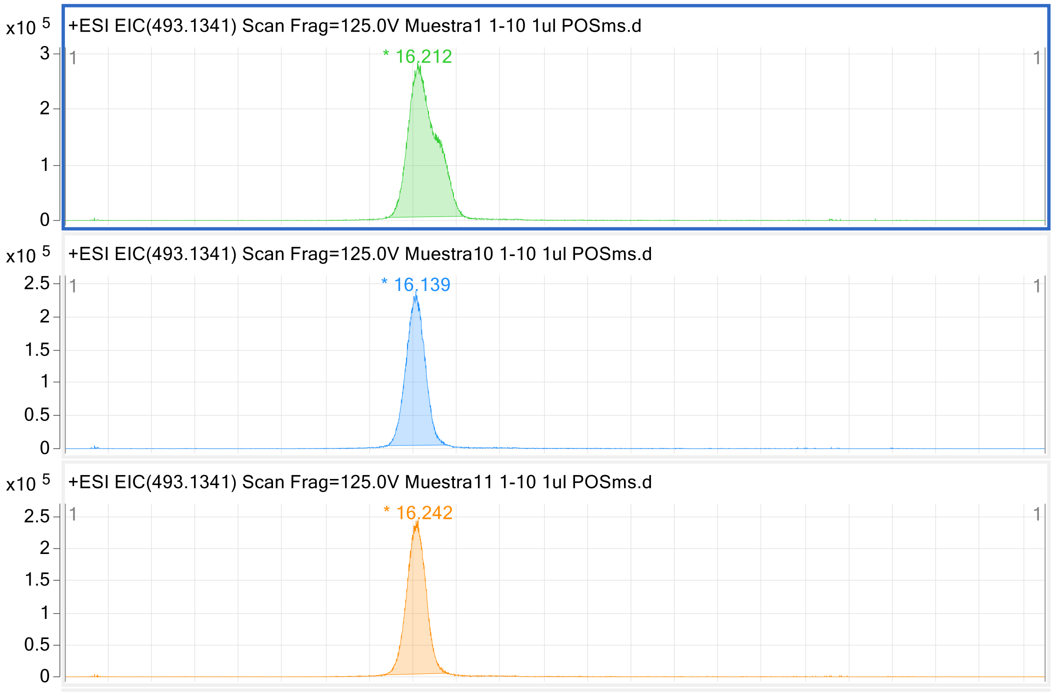

CYANIDIN-3-O-GLUCOSIDE (449.1078) IN SAMPLES

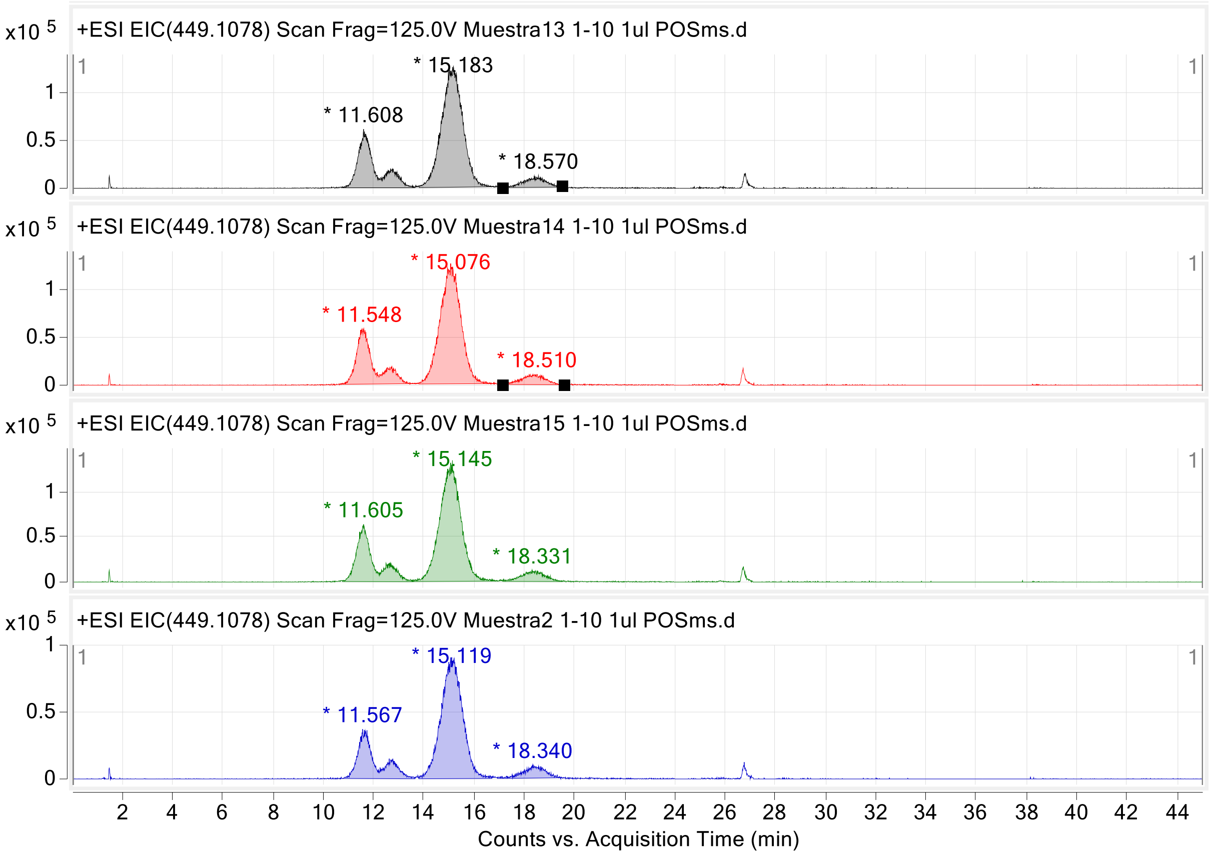

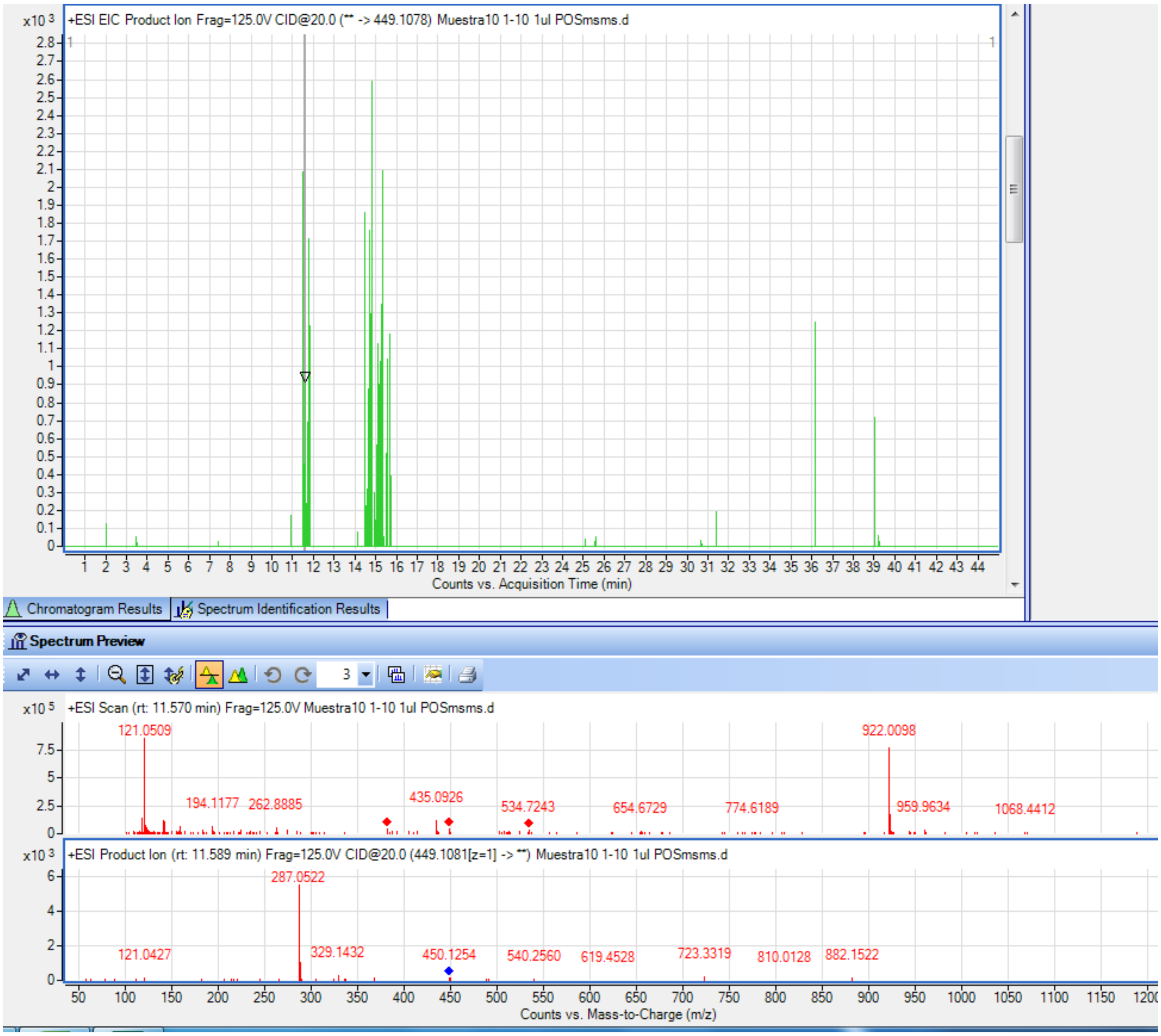
+ESI EIC Product Ion Frag=125.0V CID@20.0 (** -> 449.1078) Muestra10 1-10 1ul POSmsms.d
Counts vs. Acquisition Time (min)
Chromatogram Results
Spectrum Identification Results
Spectrum Preview
+ESI Scan (rt: 11.570 min) Frag=125.0V Muestra10 1-10 1ul POSmsms.d
121.0509
194.1177
262.8885
435.0926
534.7243
654.6729
774.6189
922.0098
959.9634
1068.4412
+ESI Product Ion (rt: 11.589 min) Frag=125.0V CID@20.0 (449.1081[z=1] -> **) Muestra10 1-10 1ul POSmsms.d
287.0522
121.0427
329.1432
450.1254
540.2560
619.4528
723.3319
810.0128
882.1522
Counts vs. Mass-to-Charge (m/z)

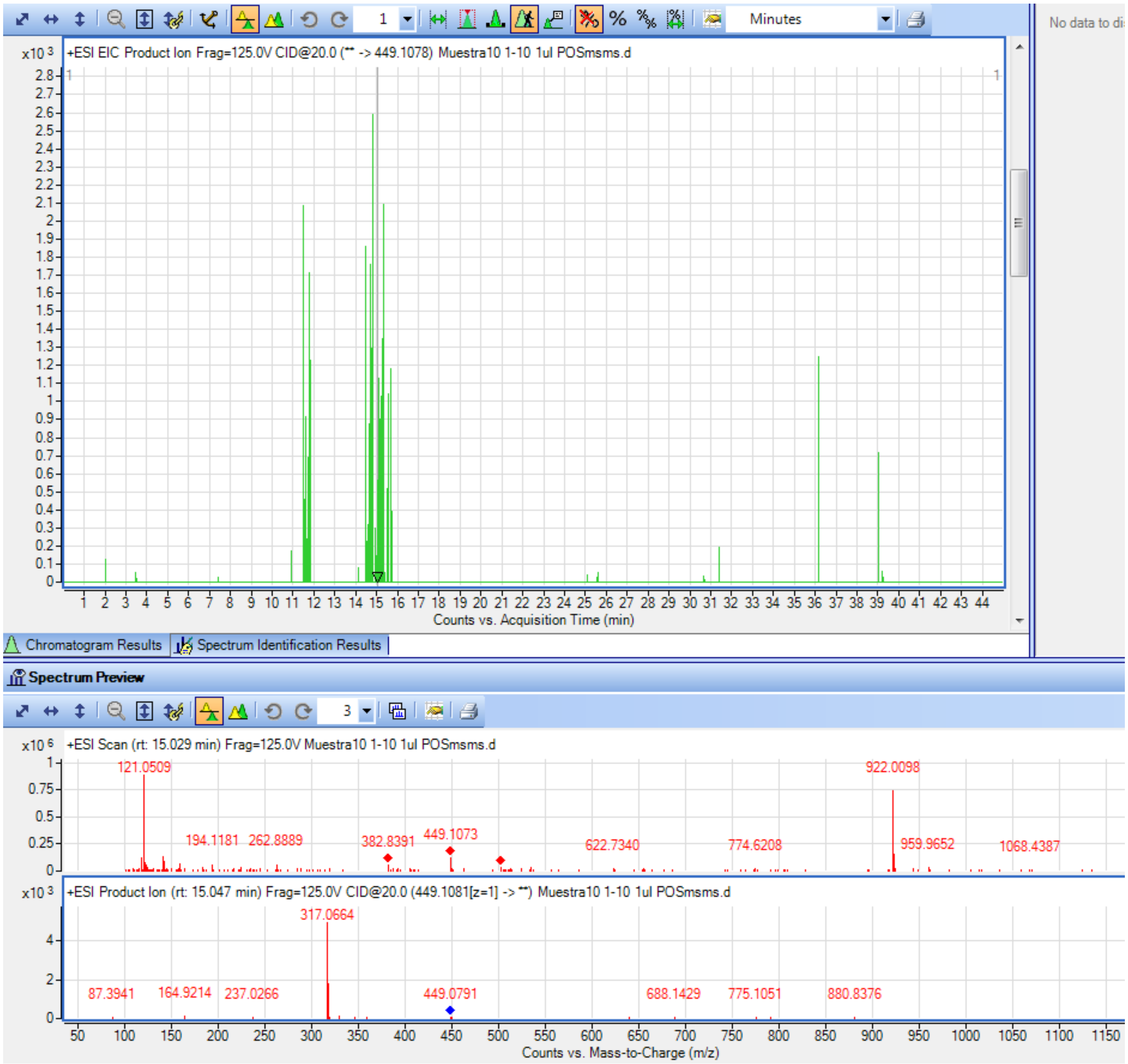

+ESI EIC Product Ion Frag=125.0V CID@20.0 (** -> 449.1078) Muestra10 1-10 1ul POSmsms.d
Counts vs. Acquisition Time (min)
Chromatogram Results
Spectrum Identification Results
Spectrum Preview
+ESI Scan (rt: 15.029 min) Frag=125.0V Muestra10 1-10 1ul POSmsms.d
121.0509
922.0098
194.1181
262.8889
382.8391
449.1073
622.7340
774.6208
959.9652
1068.4387
+ESI Product Ion (rt: 15.047 min) Frag=125.0V CID@20.0 (449.1081[z=1] -> **) Muestra10 1-10 1ul POSmsms.d
317.0664
87.3941
164.9214
237.0266
449.0791
688.1429
775.1051
880.8376
Counts vs. Mass-to-Charge (m/z)

DELPHINIDIN 3-ARABINOSIDE (435.0922) AND 3-PYRANOSIDE (465.1028) IN SAMPLES

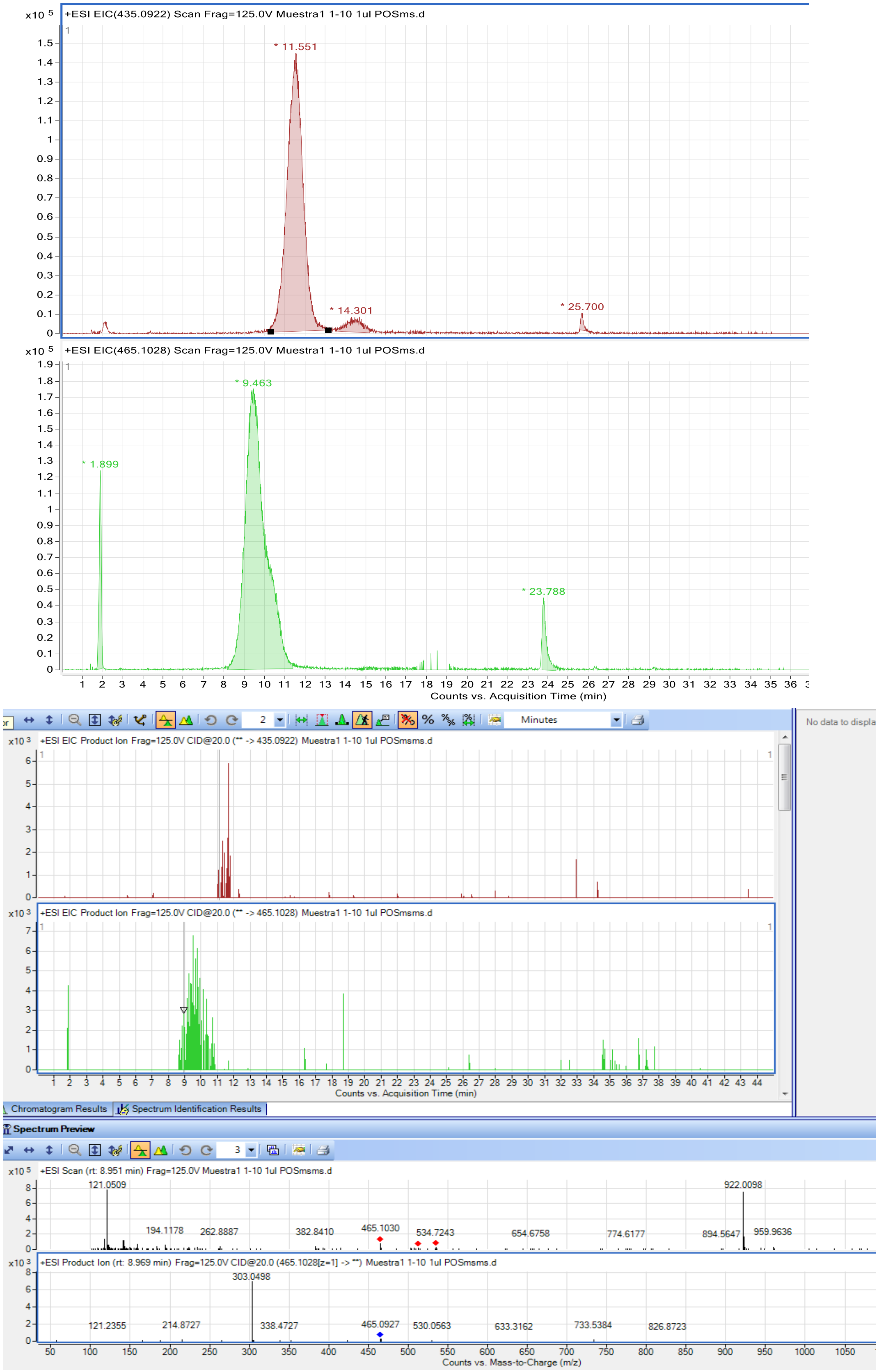

MALVIDIN-3-ARABINOSIDE (463.1235) AND PEONIDIN 3-PYRANOSIDE (463.1235) IN SAMPLES

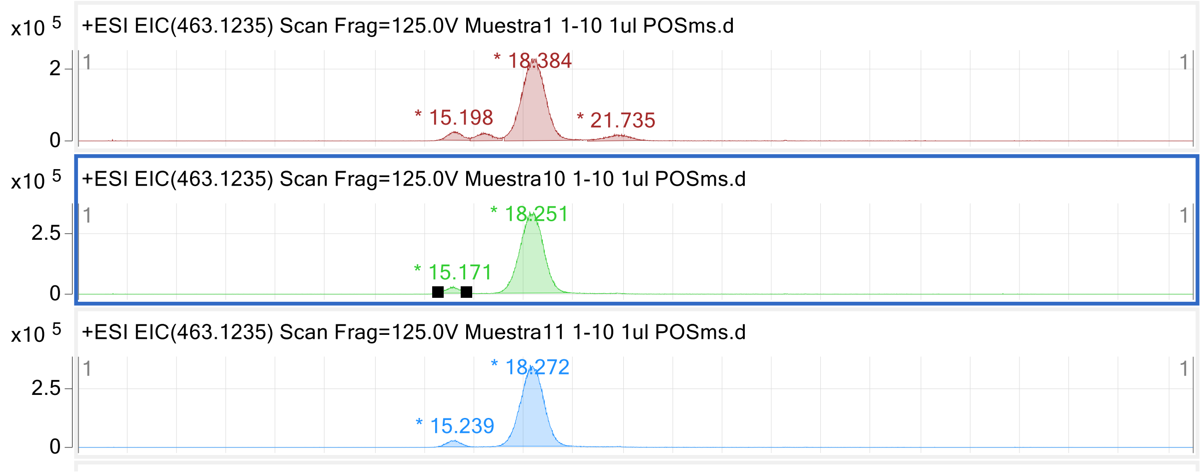

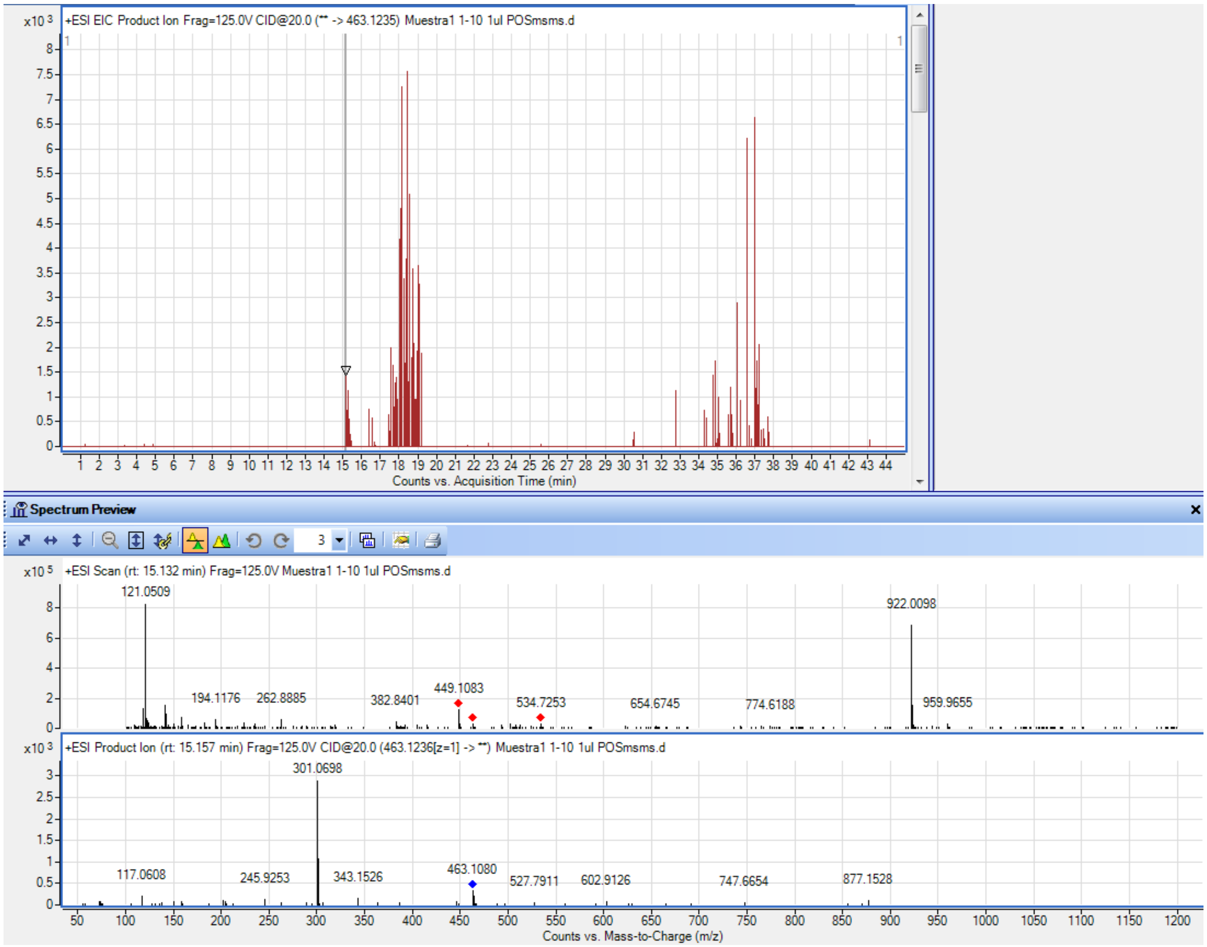

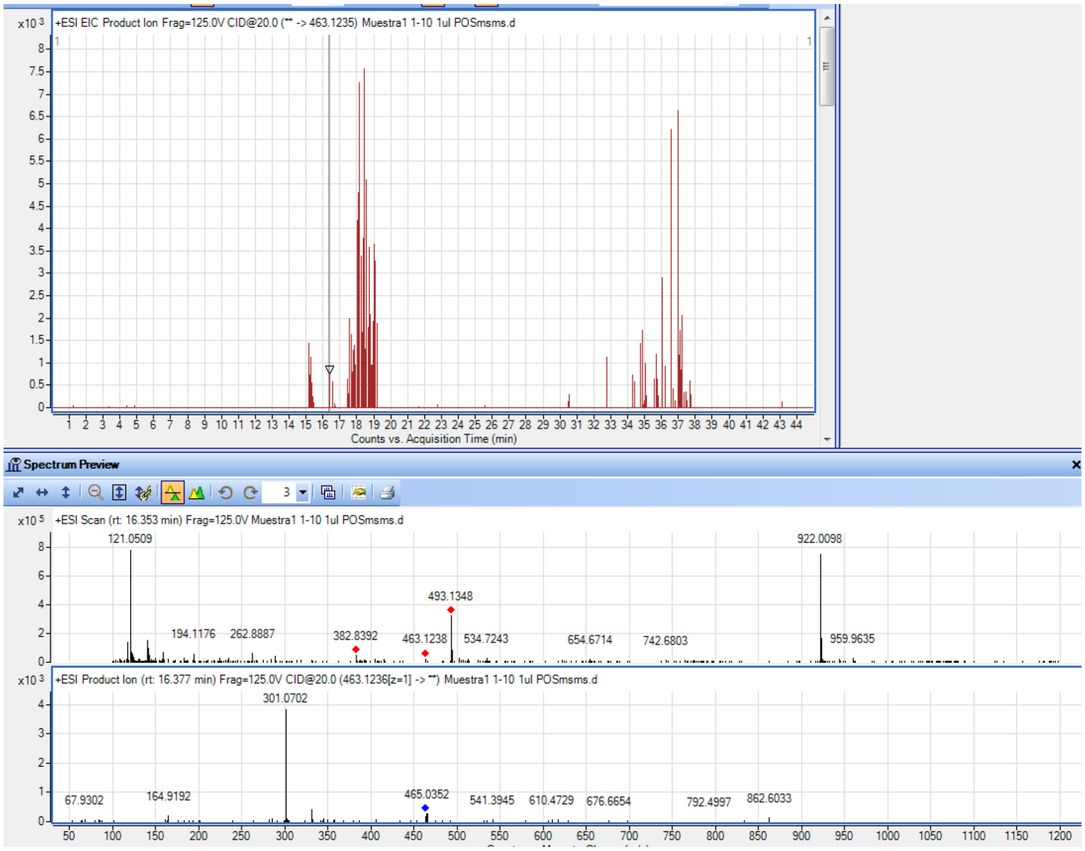
+ESI EIC Product Ion Frag=125.0V CID@20.0 (** -> 463.1235) Muestra1 1-10 1ul POSmsms.d
Counts vs. Acquisition Time (min)
Spectrum Preview
+ESI Scan (rt: 16.353 min) Frag=125.0V Muestra1 1-10 1ul POSmsms.d
121.0509
922.0098
493.1348
194.1176
262.8887
382.8392
463.1238
534.7243
654.6714
742.6803
959.9635
+ESI Product Ion (rt: 16.377 min) Frag=125.0V CID@20.0 (463.1236[z=1] -> **) Muestra1 1-10 1ul POSmsms.d
301.0702
67.9302
164.9192
465.0352
541.3945
610.4729
676.6654
792.4997
862.6033

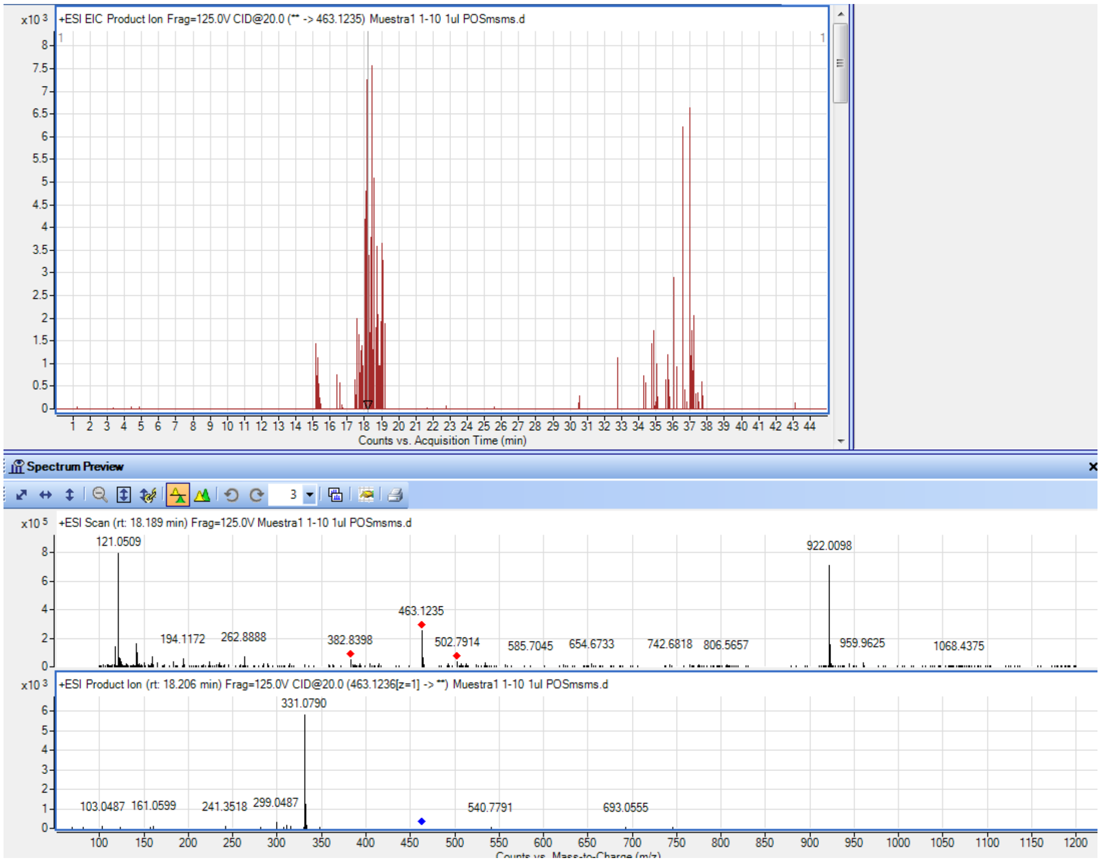
+ESI EIC Product Ion Frag=125.0V CID@20.0 (** -> 463.1235) Muestra1 1-10 1ul POSmsms.d
Counts vs. Acquisition Time (min)
Spectrum Preview
+ESI Scan (rt: 18.189 min) Frag=125.0V Muestra1 1-10 1ul POSmsms.d
121.0509
922.0098
463.1235
194.1172
262.8888
382.8398
502.7914
585.7045
654.6733
742.6818
806.5657
959.9625
1068.4375
+ESI Product Ion (rt: 18.206 min) Frag=125.0V CID@20.0 (463.1236[z=1] -> **) Muestra1 1-10 1ul POSmsms.d
331.0790
103.0487
161.0599
241.3518
299.0487
540.7791
693.0555

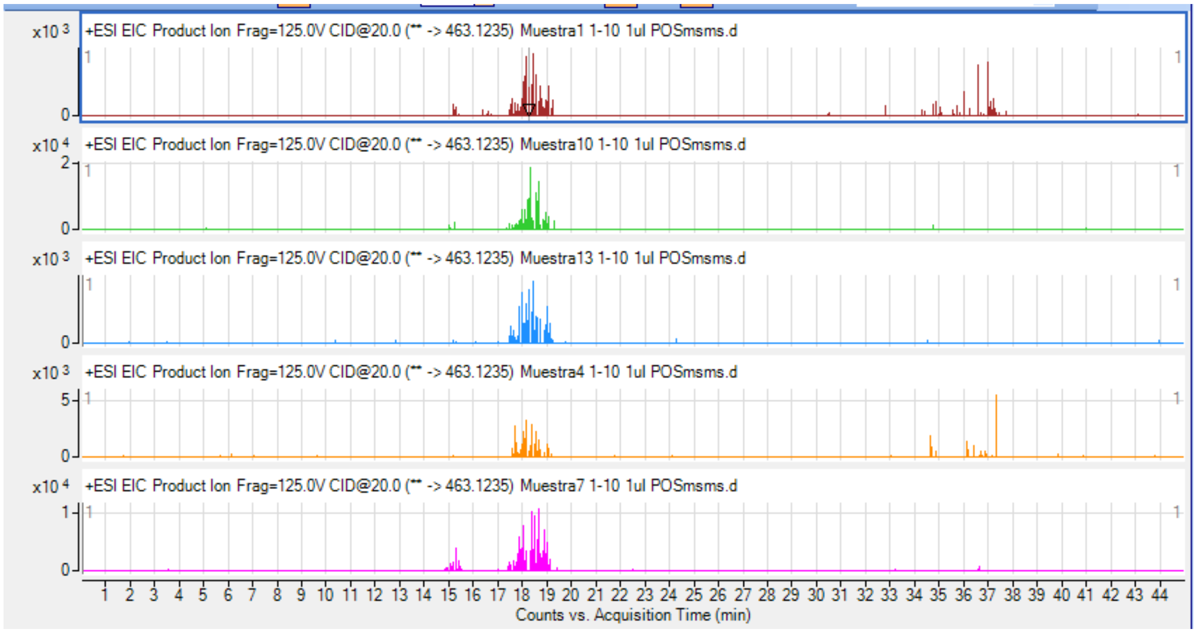

PETUNIDIN-3-PYRANOSIDE (479.1184) IN SAMPLES

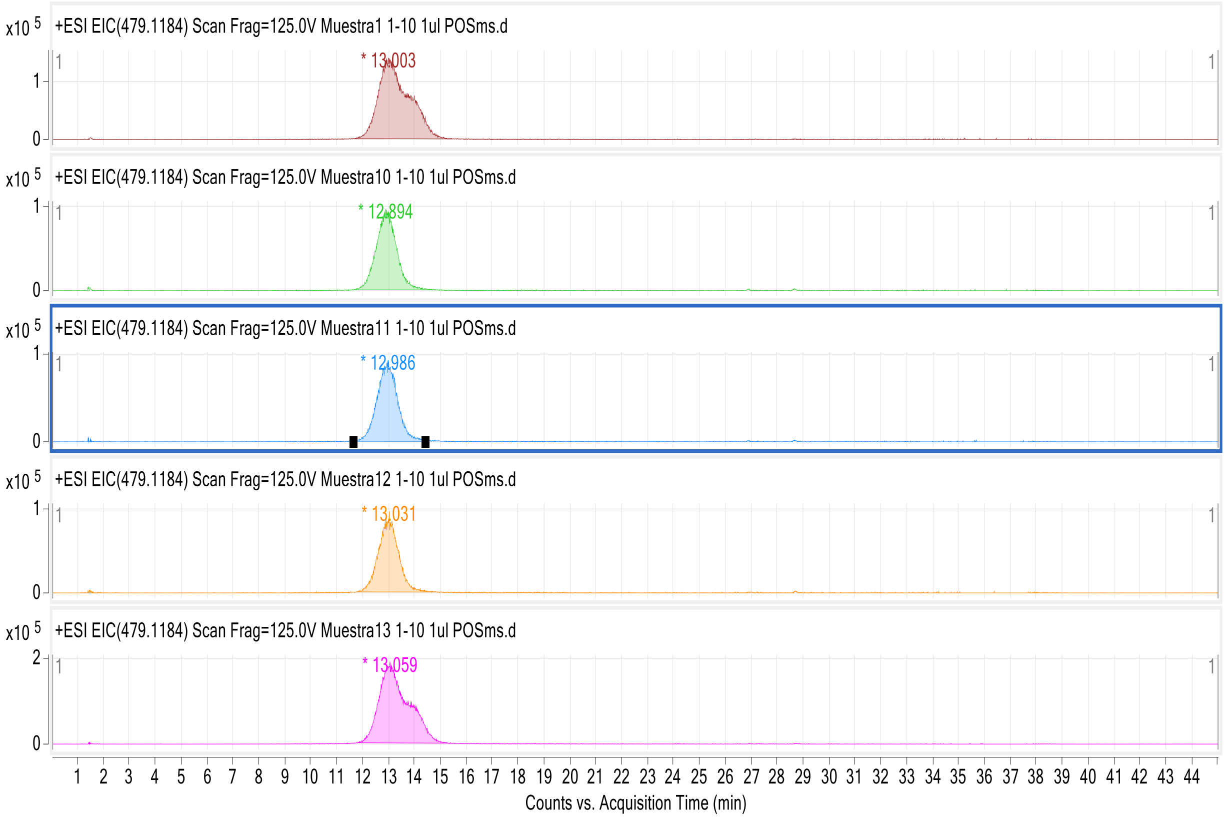

CATECHIN/EPICATECHIN (291.0863) IN SAMPLES

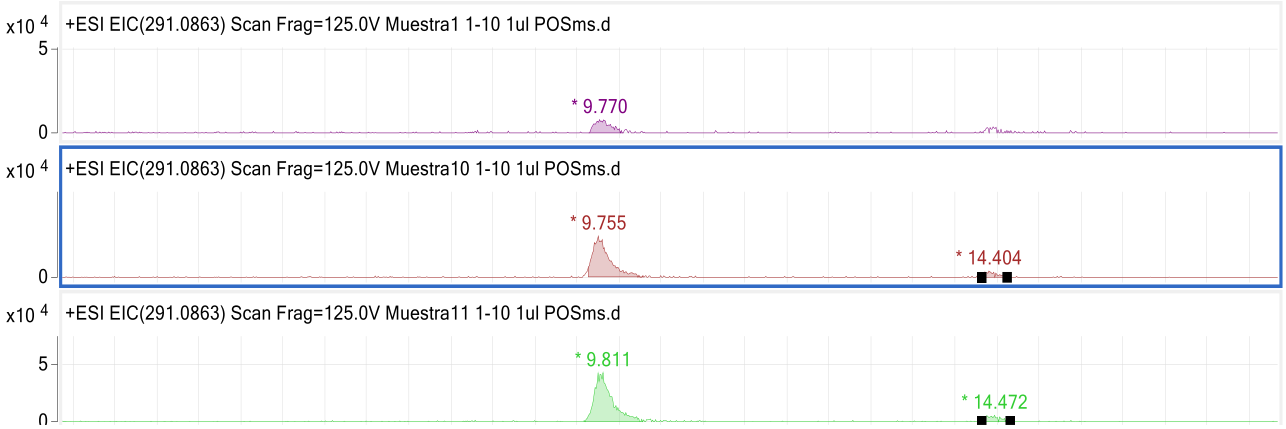

CYANIDIN 3-ARABINOSIDE (419.0973) IN SAMPLES

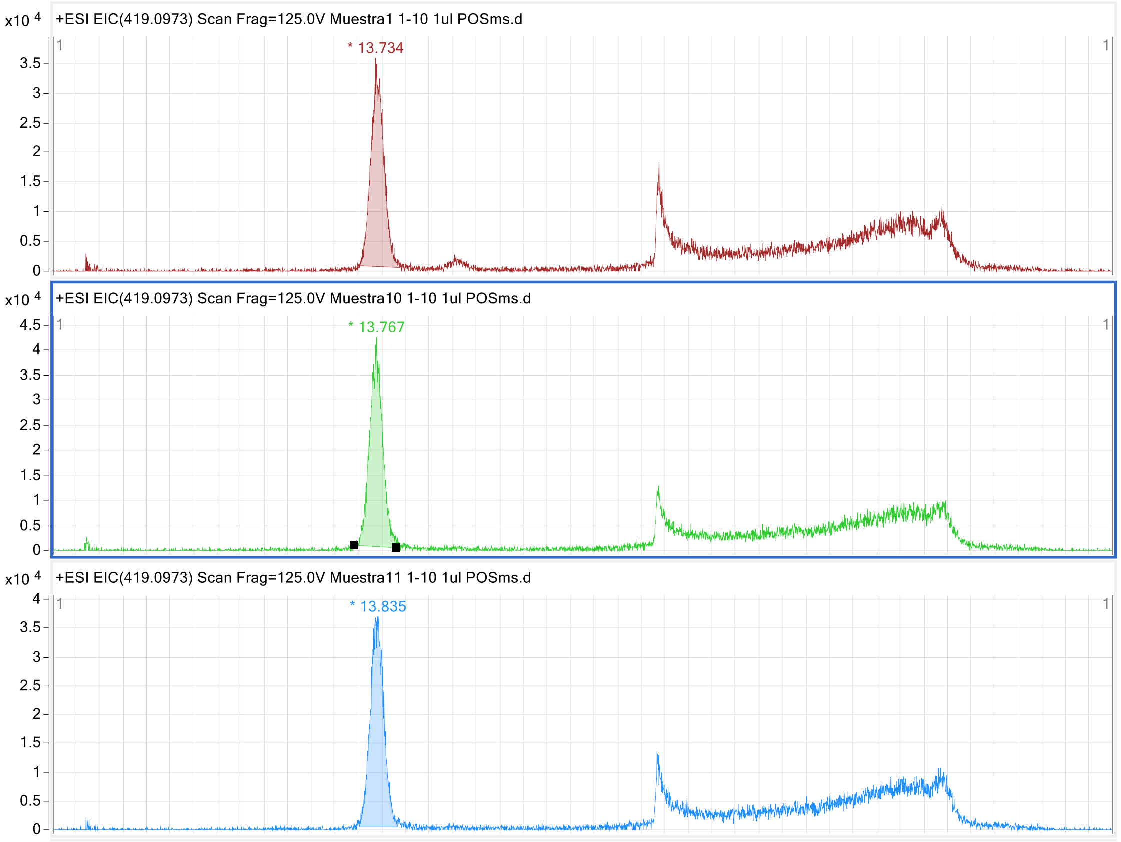

Best	ID Source	Name	Formula	Species	m/z	Score	Score (RT)	RT Diff	Diff (ppm)	Score (Lib)
	MFG		C20 H18 O10	(M+H)+	419.0972	99.1			-0.17	
	MFG		C19 H12 N7 O5	(M+H)+	419.0972	97.47			-0.55	
	MFG		C21 H14 N4 O6	(M+H)+	419.0972	94.89			2.84	
	MFG		C18 H16 N3 O9	(M+H)+	419.0972	92.38			-3.56	
	MFG		C17 H10 N10 O4	(M+H)+	419.0972	89.69			-3.97	
	MFG		C27 H16 N O2 S	(M+H)+	419.0972	88.97			-0.29	
	MFG		C25 H14 N4 O S	(M+H)+	419.0972	84.43			-3.66	
	MFG		C13 H18 N6 O8 S	(M+H)+	419.0972	84.13			0.41	
	MFG		C23 H16 N O7	(M+H)+	419.0972	84.01			6.22	
	MFG		C14 H14 N10 O4 S	(M+H)+	419.0972	82.89			3.4	
	MFG		C20 H16 N7 S2	(M+H)+	419.0972	82.84			0.75	
	MFG		C21 H22 O5 S2	(M+H)+	419.0972	82.58			1.1	
	MFG		C22 H10 N8 O2	(M+H)+	419.0972	82.25			5.88	
	MFG		C15 H20 N3 O9 S	(M+H)+	419.0972	81.05			3.86	
	MFG		C19 H20 N3 O4 S2	(M+H)+	419.0972	80.35			-2.28	
	MFG		C16 H14 N6 O8	(M+H)+	419.0972	78.15			-6.98	
	MFG		C12 H22 N2 O12 S	(M+H)+	419.0972	77.44			-2.53	
	MFG		C11 H16 N9 O7 S	(M+H)+	419.0972	76.7			-3.07	
	MFG		C24 H18 O5 S	(M+H)+	419.0972	76.42			-6.69	
	MFG		C22 H18 N4 O S2	(M+H)+	419.0972	76.21			4.13	

Chromatogram Results | Spectrum Identification Results: + Scan (rt: 13.169-14.420 min)

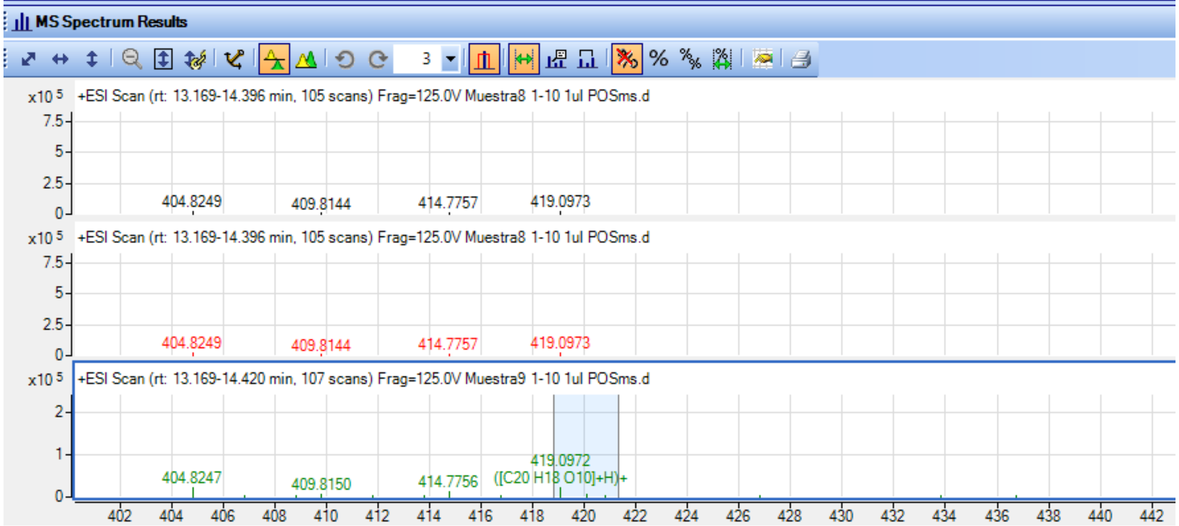

Supplement: Supplementary file 1 [file foods-12-02621-s001.zip › foods-2458571-supplementary.pdf]
